# Atopic eczema in primary care: evidence update and implications for practice

**DOI:** 10.3399/bjgp24X736101

**Published:** 2023-12-29

**Authors:** Miriam Santer, Matthew J Ridd, Jane Harvey, Stephanie Lax, Ingrid Muller, Amanda Roberts, Kim S Thomas

**Affiliations:** Primary Care Research Centre, University of Southampton, Southampton.; Centre for Academic Primary Care, University of Bristol, Bristol.; Centre of Evidence Based Dermatology, School of Medicine, University of Nottingham, Nottingham.; Centre of Evidence Based Dermatology, School of Medicine, University of Nottingham, Nottingham.; Primary Care Research Centre, University of Southampton, Southampton.; Centre of Evidence Based Dermatology, School of Medicine, University of Nottingham, Nottingham.; Centre of Evidence Based Dermatology, School of Medicine, University of Nottingham, Nottingham.

Most people with eczema have mild or moderate disease, and most are treated in primary care.^1^ This article aims to support health professionals in helping patients get control of eczema in time-limited consultations. Recent updated National Institute for Health and Care Excellence (NICE) guidance on atopic eczema highlighted changes to advice regarding bath emollients and advice on how to wash.^2^ While a fuller update is awaited, the evidence behind this is presented here, along with a summary of other recent research on eczema.

## Signposting to self-management website improves eczema outcomes

For many people with eczema the main barrier to treatment control is effective use of emollients and topical corticosteroids (TCS).^3^ Understanding the different roles of these two treatments is crucial: *topical corticosteroids get control while emollients keep control of eczema*. Recent research has shown that a freely available website (Eczema Care Online; https://www.eczemacareonline.org.uk) supporting eczema self-management leads to improved eczema outcomes for both children and young people.^4^

**Figure 1.**
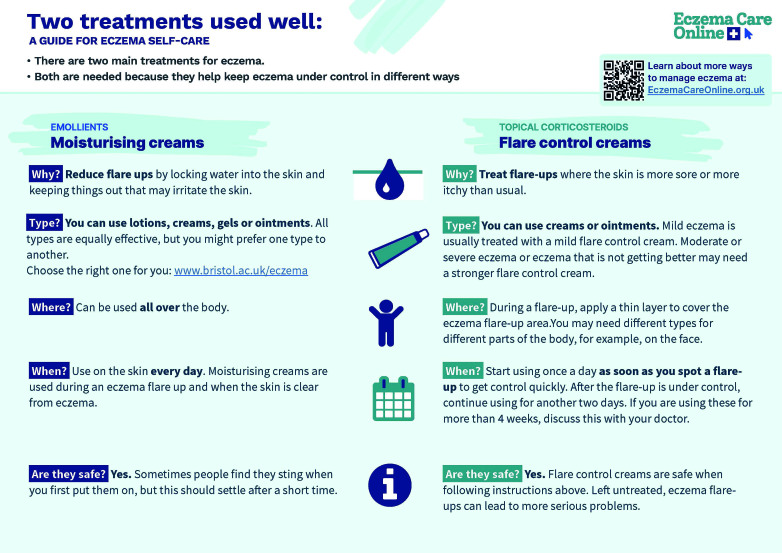
Printable infographic for patients to promote the different roles of emollients, *‘moisturising creams’*, and topical corticosteroids, *‘flare control creams’.* Source: www.eczemacareonline.org.uk/en/two-treatments-documents. © The University of Southampton. Used with permission.

Eczema Care Online was developed together with patients/ carers and incorporated extensive user feedback. This showed that terminology can be a barrier to treatment use, as *emollient* sounds ‘medical’ (therefore people do not like using it long term) and *steroid* has negative associations. Treatment may be better understood by using the terms *‘flare control creams’* to get control and *‘moisturising creams’* to keep control. User feedback also showed that use of *‘finger-tip units’* added to uncertainty around TCS, and users preferred *‘put on a thin layer, just enough to cover the eczema flare area’* (Figure 1).

## How to prescribe topical corticosteroids safely and effectively

Patients and carers report concerns over the safety of TCS, particularly for long-term use.^5^ These concerns can be heightened by inconsistent messaging from healthcare professionals.^6^ A recent Cochrane systematic review provided reassuring data around the safety and effectiveness of different strategies for using TCS to help provide consistency.^7^ A further review that included observational studies, as well as the randomised controlled trials (RCTs) in the Cochrane review, showed that, although data on longer-term safety of TCS are scarce, studies with follow-up longer than a year are reassuring.^8^

### Safety

The Cochrane review supports recommendations that TCS are safe when *‘used appropriately’*, that is, for up to 4 weeks, depending on potency, site, and eczema thickness, then having a break to minimise potential side effects. Skin thinning was reported in less than 1% of participants included in studies within the review, mostly occurring with use of very potent TCS.^8^

### Once-daily topical corticosteroid

There is no evidence of a difference in effectiveness between twice-daily application versus once-daily application of TCS in eczema.^8^ This supports previous recommendations advising using TCS once daily, to simplify treatment regimens, and potentially minimise adverse events compared with twice-daily TCS.^9^^,^^10^

### Which potency of topical corticosteroid to treat a flare?

Among people with moderate eczema, or worse, there is good evidence that moderate and potent TCS are likely to be more effective in treating eczema flares compared with mild TCS.^8^ Studies in people with mild eczema are lacking.^8^

### ‘Weekend therapy’ of topical corticosteroid to prevent recurrent flares

For people who have frequent eczema flares despite use of regular emollients, there is good evidence that applying the TCS to areas prone to eczema 2 consecutive days per week (‘weekend’ or ‘proactive’ therapy) prevents flare-ups. It also appears to be safe as no cases of skin thinning were reported in the trials of this strategy.^7^

## How to prescribe emollients effectively

### Leave-on emollients

Acceptability and perceived effectiveness of emollients are key to their being used regularly to improve eczema symptoms. There are many different products, but most (from thin through to thick consistency) are lotions, creams, gels, or ointments.

Recent evidence in childhood eczema has shown that all emollient types are similarly effective, contradicting the previous consensus that ‘thicker’ emollients need to be applied less often and are better for more severe eczema.^11^ Awareness of the different emollient types is low and different types suit different people, that is, the best emollient is the one that the patient will use.

Localised skin reactions are common with all types, affecting around a third of patients. As emollients are more likely to sting when eczema is not well controlled, this may reflect under-use of TCS. An emollient decision aid, which summarises the different types, is available to download from https://www.bristol.ac.uk/eczema.

### Bath emollients and washing with eczema

Bath emollient additives do not add benefit when used in addition to leave-on emollients for childhood eczema,^12^ further simplifying treatment regimens. People with eczema should be advised to avoid using soaps, reduce shampoo contact with the skin, and use leave-on emollients as soap substitutes. Eczema Care Online has clear advice on how to wash with eczema.

## Putting it all together

There is a lot of information to be conveyed within an eczema consultation, including potential triggers, understanding treatments (‘flare control creams’ to get control and ‘moisturising creams’ to keep control), and how to wash. Using high-quality free resources such as Eczema Care Online is a time-efficient way of delivering key messages and improving outcomes for eczema (Box 1).

**Box 1.** Eczema resources

**Resource**	**URL**	**Description**
Eczema Care Online	https://www.eczemacareonline.org.uk	Evidence-based website for people with eczema and parents of babies or children with eczema
‘Two treatments used well’ printable leaflet	https://www.eczemacareonline.org.uk/en/two-treatments-documents	Summarises *‘flare control creams’* get control and *‘moisturising* *creams’* keep control
Moisturiser Decision Aid	https://www.bristol.ac.uk/eczema	Designed for parents, older children with eczema, or health professionals, to inform emollient choice
Eczema Written Action Plan	https://www.bristol.ac.uk/eczema	Patient-held plan, to be completed with health professional, for parents of children with eczema

## References

[1] de Lusignan S, Alexander H, Broderick C (2021). Patterns and trends in eczema management in UK primary care (2009–2018): a population-based cohort study. Clin Exp Allergy.

[2] Matthews SJ, Housam N, Lawton S (2023). Atopic eczema in under 12s: diagnosis and management — summary of updated NICE guidance. BMJ.

[3] National Institute for Health and Care Excellence (2023). Atopic eczema in under 12s: diagnosis and management CG57.

[4] Santer M, Muller I, Becque T (2022). Eczema Care Online behavioural interventions to support self-care for children and young people: two independent, pragmatic, randomised controlled trials. BMJ.

[5] Hon KL, Tsang YCK, Pong NH (2015). Correlations among steroid fear, acceptability, usage frequency, quality of life and disease severity in childhood eczema. J Dermatol Treat.

[6] Teasdale E, Muller I, Sivyer K (2021). Views and experiences of managing eczema: systematic review and thematic synthesis of qualitative studies. Br J Dermatol.

[7] Lax SJ, Harvey J, Axon E (2022). Strategies for using topical corticosteroids in children and adults with eczema. Cochrane Database Syst Rev.

[8] Harvey J, Lax SJ, Lowe A (2023). The long-term safety of topical corticosteroids in atopic dermatitis: a systematic review. Skin Health Dis.

[9] Williams HC (2007). Established corticosteroid creams should be applied only once daily in patients with atopic eczema. BMJ.

[10] Green C, Colquitt JL, Kirby J, Davidson P (2005). Topical corticosteroids for atopic eczema: clinical and cost effectiveness of once-daily vs. more frequent use. Br J Dermatol.

[11] Ridd MJ, Santer M, MacNeill SJ (2022). Effectiveness and safety of lotion, cream, gel, and ointment emollients for childhood eczema: a pragmatic, randomised, phase 4, superiority trial. Lancet Child Adolesc Health.

[12] Santer M, Ridd MJ, Francis NA (2018). Emollient bath additives for the treatment of childhood eczema (BATHE): multicentre pragmatic parallel group randomised controlled trial of clinical and cost effectiveness. BMJ.

